# Histomorphology of placentae of women with sickle cell disease during pregnancy – A case control study

**DOI:** 10.1371/journal.pone.0319011

**Published:** 2025-02-24

**Authors:** Mohammed Mumuni, Kevin Kofi Adutwum-Ofosu, Benjamin Arko-Boham, Bismarck Afedo Hottor, Nii Koney-Kwaku Koney, Kwame Adu-Bonsaffoh, Samuel Antwi Oppong, Peter Ofori Appiah, John Ahenkorah

**Affiliations:** 1 Department of Anatomy, University of Ghana Medical School, College of Health Sciences, University of Ghana, Korle Bu Campus, Accra, Ghana; 2 Department of Obstetrics and Gynaecology, University of Ghana Medical School, College of Health Sciences, University of Ghana, Korle Bu Campus, Accra, Ghana; 3 Department of Medical Microbiology, University of Ghana Medical School, College of Health Sciences, University of Ghana, Korle Bu Campus, Accra, Ghana; King Saud University/Zagazig University, EGYPT

## Abstract

**Background:**

Sickle cell disease (SCD) is known to exert multifaceted effects on pregnancy, potentially influencing placental structure and function.

**Aim:**

Our aim was to utilize stereology as a precise analytical tool to evaluate the histo-morphologic and functional changes in term placentae of women with SCD against those of non-SCD women.

**Method:**

A case control study was conducted at the Korle-Bu Teaching Hospital’s labour unit and included 38 pregnant women, comprising 19 cases and 19 controls. Placenta samples were paired and matched with gestational age and taken at term (38 weeks + 2 weeks). Tissue sections were prepared, stained with hematoxylin and eosin, and volume densities of syncytial knots, foetal capillaries, syncytial denuded areas, and intervillous spaces estimated by stereological methods. Statistical analysis was performed to compare mean values between the SCD and control groups.

**Results:**

Among the study participants with SCD, 13.16% (5) had sickle cell haemoglobin S (HbSS), 34.21% (13) had haemoglobin C (HbSC) and 2.63% (1) had β-thalassemia (HbS). On stereological assessment, there were statistically significant differences in mean volume densities of syncytial knots (*p* = < 0.0034), foetal capillaries (*p* = < 0.0001), syncytial denudations (*p* = < 0.0028), and intervillous space (*p* = < 0.0113) between term placentae of women with SCD and those without SCD.

**Conclusion:**

SCD placentae may result in a substantial increase in syncytial knot formation, possibly because of hypermaturation of the chorionic villi, significant increase in foetal capillaries potentially due to the hypoxic nature of the SCD placentae, syncytial denuded areas as a result of alteration of the placental syncytium and reduced intervillous spaces which may be due to villous congestion. These findings suggest the need for heightened monitoring of placental function and fetal well-being in pregnancies complicated by SCD to reduce adverse perinatal outcomes.

## 1. Introduction

Pregnancy is accompanied by physiological changes in the body, and the presence of genetic disorders can further make it unbearable and discomforting [1]. Sickle cell disease (SCD) is a common genetic haemoglobinopathy resulting in persistent haemolytic anaemia and vaso-occlusive crises [1]. Development of the placenta is affected by anaemia and low haemoglobin concentration and is associated with an increased risk of low-birth-weight babies, growth restriction, foetal demise, and prematurity [2–5]. Maternal consequences include deteriorating anaemia and increased vulnerability to pain crises, acute chest syndrome, and infections [6,7], leading to higher morbidity and mortality [4,8]. Globally, there are approximately 300,000 babies born with SCD annually, with the estimates predicted to reach 400,000 by the year 2050 [9,10]. Despite the increasing focus on obstetric challenges in SCD, there’s still a notable gap in the understanding of histomorphological alterations within the chorionic villi of term placentae in affected mothers which may have negative repercussions on the intrauterine development of the child.

The chorionic villi are essential for maternal-foetal and foetal-maternal exchanges, as they facilitate the placenta’s metabolic and endocrine functions [11]. These structures receive blood from both the foetal and maternal circulations, allowing the placenta to produce and secrete hormones, cytokines, growth factors, and other bioactive products crucial for foetal development. Although there is diversity in villous forms, all chorionic villi have the same fundamental structure [12,13]. As a result, any injury to the chorionic villi potentially impairs its integrity and compromises the placental barrier structures such as the syncytiotrophoblast, trophoblast basement membrane, cytotrophoblast, and foetal capillary endothelium. Although the adverse effects of SCD on pregnancy outcomes are well recognized, the contribution of placental pathology to these events remains controversial. Investigating the effects of sickle cell disease (SCD) on the histomorphology of chorionic villi in term placentae is essential for understanding the associated alterations in the micro-architecture of the placental barrier. This study focused on four key morphological features of the placenta: syncytial knots, syncytial denuded areas, foetal capillaries, and intervillous gaps, as these features are strongly associated with placental efficiency in nutrient and oxygen transfer, as well as the structural adaptations necessary to meet increased metabolic demands during pregnancy [12]. The aim of the study was to utilize stereology as a precise analytical tool to evaluate the histo-morphologic and functional changes in term placentae of women with SCD compared to those of non-SCD women. Through a better understanding of these alterations, we hope to contribute to the growing body of knowledge related to the effects of SCD on placenta histomorphology, thus providing insights for improved clinical management and in developing an effective intervention for the control of placental pathology associated with this disease.

## 2. Materials and methods

### 2.1. Study design, site and sampling

The research study was a case control study conducted at the Korle-Bu Teaching Hospital’s Labour Unit. The Korle-Bu Teaching Hospital serves as a referral centre for other health facilities throughout Ghana. The hospital’s 360-bed capacity Department of Obstetrics and Gynaecology has an average of 900 births per month and delivers 20-30 babies per day.

Sampling was by total enumeration where all consenting sickle cell pregnant mothers’ delivery at the study site during the study period were included. Each recruited SCD pregnant mother was gestational age-matched with a non SCD pregnant mother as control. The study was conducted at the Labour Unit of Department of Obstetrics and Gynaecology, Korle-Bu Teaching Hospital (KBTH) between 20^th^ of June and 25^th^ November 2022. Written informed consent was obtained from expectant pregnant women who took part in the study at the Labour Unit prior to delivery, using English, or two commonly spoken local languages (Twi or Fante). Placentae from participants were collected on site after delivery in the hospital by either spontaneous vaginal delivery (SVD) or Caesarean section (CS). Gestational age was determined based on the participants’ last menstrual periods and as recorded from their clinical folders. Once delivered, each placenta was placed in a sterile kidney dish and was then transferred into a wide stainless plastic bowl for examination. The method of placental examination was adopted from Kaplan [14].

### 2.2. Tissue sampling and processing

Each placenta was cut into four equal quadrants and by choosing a random starting point, a tissue was sampled full-depth (from chorionic plate to basal plate). Placenta samples (about 2 cm in length × 2 cm in width × 5 cm in thickness) were fixed by immersion in 10% phosphate-buffered formalin for 48 hours with a pH of 7.24–7.2.

Sampled placenta tissues were placed in tissue cassettes and processed in an automated tissue processor (Leica TP 1020, Germany). Samples were passed through ascending grades of ethanol (from 70% to 100%) for dehydration, and then transferred into two changes of xylene for an hour for clearing. The tissues were embedded in molten wax at (58°C), moulded into blocks and then prepared for sectioning by microtomy.

### 2.3. Sectioning of placenta tissue and staining

The placenta tissue blocks were trimmed at 9 µm using a microtome (Leica RM 2125, Wetzlar, Germany) to expose the whole profiles of the tissues and then sectioned at 5 µm. Three sections were selected per tissue block. The 1^st^ section was randomly selected after the tissue was exposed, then the 50^th^ and 100^th^ sections were systematically selected. From one placenta, a maximum of 12 tissue sections were selected. Sections were stained with hematoxylin and eosin (H&E) and mounted for assessment and histomorphometric quantification.

### 2.4. Sampling of photomicrographs of placentae sections

**Fig 1:**
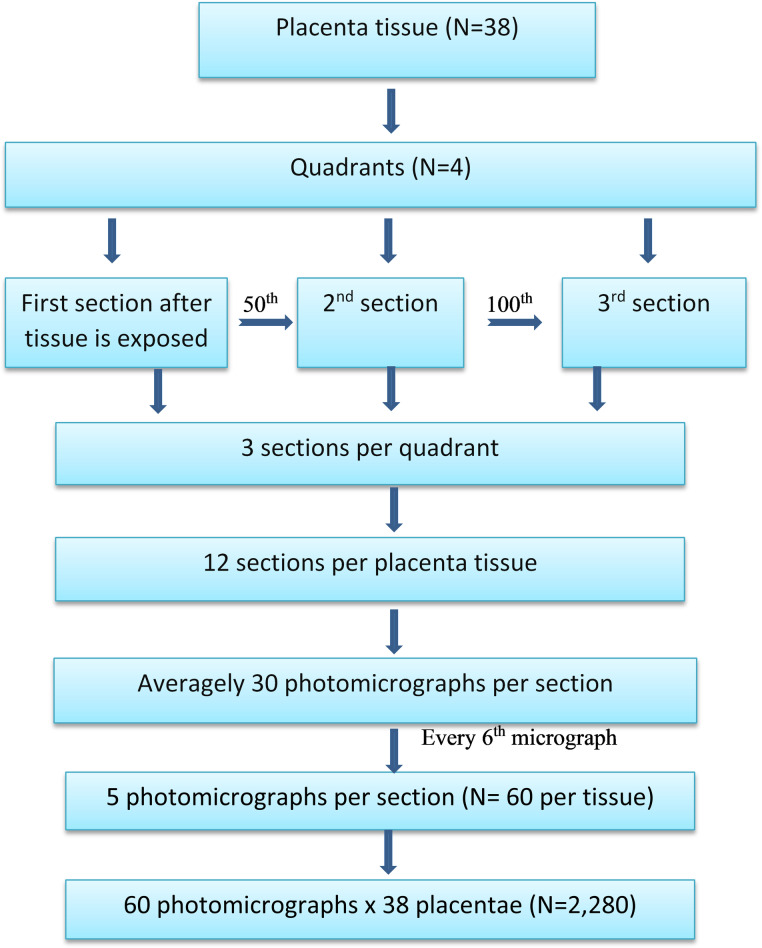
Flow chart of tissue sampling using systematic uniform random sampling to obtain micrographs. (Boxes) Process steps, (Arrows) Directional flow of the processes, (N) Number of samples.

Photomicrographs of placenta sections were obtained using an optical light compound microscope (Leica Galen III, catalogue e no. 317506, serial no. 1125DP) connected to a desktop computer (HP Compaq dx2300 Microtower) via a digital microscope eyepiece (Lenovo Q350 USB PC Camera). Movement of the stage of the microscope was 3 X 3 graduation units on the X and Y axes. The movement of the microscope stage was done on one plane in the X axis and another plane in the Y axes. A x40 objective lens with a camera (Lenovo Q350 USB PC Camera) was used to take photomicrographs on both the X and Y axes. This was done repeatedly until the whole placenta section was covered. An average of 30 micrographs were obtained from each section. Out of the about 30 photomicrographs taken from a section, five (5) were selected using systematic uniform random sampling where every 6^th^ micrograph was selected from the first randomly selected photomicrograph for stereological analysis. A total of sixty (60) photomicrographs were selected systematically from a placenta as illustrated in (Fig 1). A total of two thousand, two hundred and eighty (2,280) photomicrographs were selected systematically for the quantification of counting syncytial knots, syncytial necrosis, syncytial denudated areas, intervillous space, and foetal capillaries.

### 2.5. Stereological examination of placental photomicrographs

The mean volume densities of syncytial knots, syncytial denuded areas, foetal capillaries, and intervillous gaps were estimated using Cavalieri’s point counting approach. Adobe Photoshop CS6 Extended software (trial version 13.0.1) was used to superimpose a stereological grid of uniformly spaced points (1 cm × 1 cm) on each photomicrograph of a placenta section. The number of tests at the grid intersections were counted. Values from the point counting were entered into the formula (Cavalieri estimator of volume) below for the calculation of relative volume densities.

Vv = (a/pƩP) × t/M^2^

Where ƩP is the sum of all test points encountered, t is the thickness of the section, Vv indicates volume density, (a/p) is the area per point of the stereological grid, and M is the linear magnification.

### 2.6. Statistical analysis

Data were entered into Microsoft Excel 2019 (version 16, Microsoft Corporation, Washington, DC, USA) and analysed using GraphPad Prism software (Version 5, GraphPad Software LLC, Boston, MA, USA). Results of this study are presented as means, and standard errors of the means (SEMs), with 95% confidence intervals (CIs) for means. One-way analysis of variance (ANOVA) and *t*-tests were conducted to compare mean values within and between groups. *P*-values less than 0.05 were considered statistically significant. Bartlett’s test of equality of variances was used to test for the homogeneity of variance.

### 2.7. Ethical consideration

This work was conducted in accordance with the Declaration of Helsinki (1964). The research study was approved by the Ethical and Protocol Review Committee of the College of Health Sciences, University of Ghana with protocol identification CHS-Et/M.7-P5.5/2021-2022.

### 2.8. Consent

Written informed consent was obtained from participants prior to participation in the study.

## 3. Results

### 3.1. Distribution of sickle cell genotypes among participants

Thirty-eight placentae were collected within the study period. This comprised placentae from 19 pregnant women with SCD (cases), and 19 pregnant women without SCD (controls) who delivered at full term (38 ± 2 weeks). Of the 19 SCD participants, 5 (13.16%) had sickle cell haemoglobin S (HbSS), 13 (34.21%) had haemoglobin C (HbSC) whilst 1 (2.63%) had β-thalassaemia (HbS) as shown in Table 1 below.

**Table 1:** Sickle cell genotypes of participants.

Category	Sickle cell genotypes	Total n (%)
**Cases**	Sickle cell haemoglobin S (HbSS)	5 (13.16%)
	Sickle cell haemoglobin C (HbSC)	13 (34.21%)
	β-thalassaemia (HbS)	1 (2.63%)
**Controls**		19 (50%)
**Total**		**38 (100%)**

### 3.2. Placental histomorphological parameters

**Fig 2:**
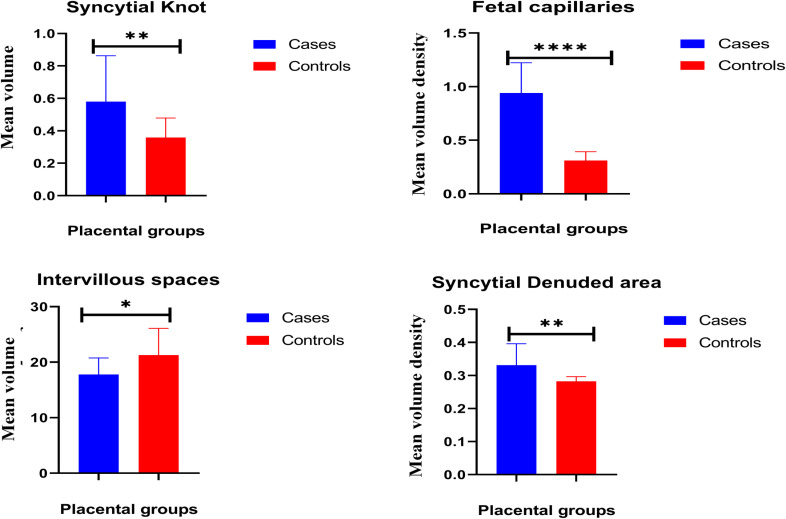
Bar chart analyses of term placental parameters. (A) Volume density of syncytial knots. (B) Volume density of foetal capillaries (C) Volume density of intervillous space. (D) Volume density of syncytial denudation. Values are expressed as mean ± SEM. P-value represents significance level for unpaired t-test for time course assessment for term group comparison with * = p < 0.05, ** = p < 0.01, *** = p < 0.001.

Unpaired t-test analyses of the placentae at term showed significant differences between the cases group and the control group (Fig 2). The volume densities of syncytial knots, foetal capillaries, and of syncytial denuded areas were significantly higher in the cases group (Fig 2). However, the mean volume density of intervillous space was significantly lower in the cases group. The SCD placentae showed a significantly higher mean volume density of syncytial knots (0.58 ± 0.065) compared to controls (0.3589 ± 0.02755, p < 0.0034). Syncytial denudation was also higher in SCD placentae (0.3311 ± 0.01492) compared to controls (0.2821 ± 0.003296, p < 0.0028) (Table 2). Also, the volume density of fetal capillaries was substantially increased in the SCD group (0.9411 ± 0.06468) compared to controls (0.31 ± 0.01906, ****p < 0.0001) (Table 2). Conversely, the intervillous spaces were significantly reduced in the SCD group (17.8 ± 0.6794) compared to

**Table 2:** Volume density of placental parameters in the various placental groups. The differences between the mean volume densities were statistically significant (higher) between the placental groups (Table 2).

Placental groups	Syncytial knots	Intervillous spaces	Syncytial denudation	Fetal capillaries
Control (n = 19)	0.3589 + 0.02755	21.27 ± 1.107	0.2821 ± 0.003296	0.31 ± 0.01906
SCD (n = 19)	0.58 + 0.06500	17.8 ± 0.6794	0.3311 ± 0.01492	0.9411 ± 0.06468
	**	*	**	****
P-value	< 0.0034	< 0.0113	< 0.0028	< 0.0001

Values are shown as mean ± SEM (standard error of the mean) with p values representing the level of significance for one-way analysis of variance (followed by Tukey’s post hoc) with * = p < 0.05, ** = p < 0.01, *** = p < 0.001 compared to the control.

**Fig 3:**
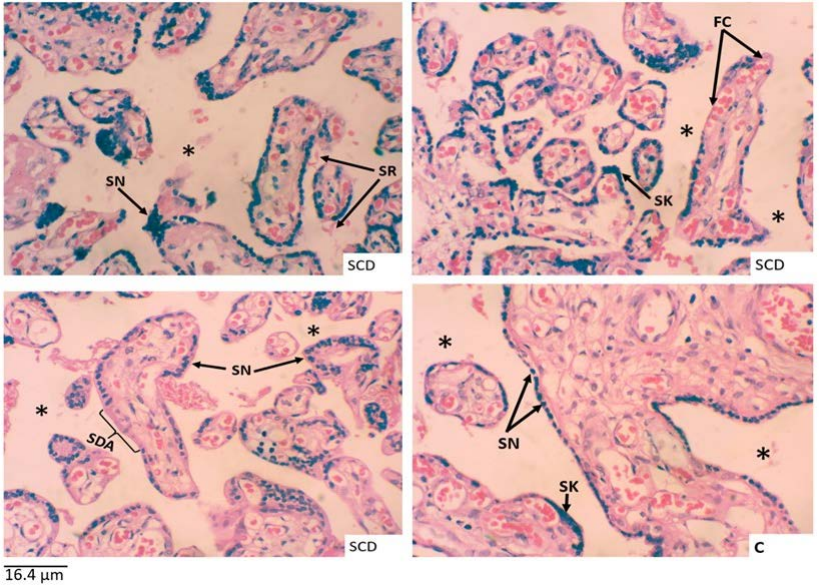
Histomorphology of H&E stained placental tissues. (C) Placental tissue from control sample with normal syncytium. (SCD) Placental tissue from cases. KEY: SN = syncytial nuclei, SK = syncytial knots, SR = sickle cell shaped RBCs, SDA = syncytial denuded area, FC = foetal capillaries, Asterisk (*) = intervillous spaces.

Though syncytial knots were recorded in both cases and controls, the clumps of syncytiotrophoblast nuclei forming the syncytial knots were significantly more common in placentae of SCD mothers than those of non-SCD mothers (Fig 3). Additionally, foetal capillaries and syncytial denuded areas were significantly increased in SCD placentae compared to controls (Fig 3). The volume density of intervillous space was much lower in the SCD cases than in controls.

## 4. Discussion

The present study compared the effect of SCD on placental histomorphology. Syncytial knot formation was observed in both cases and controls in this study. A study has suggested that, prior to 32 weeks of gestation, syncytial knots are uncommon; nevertheless, as gestation advances, they increase in number [15]. This might demonstrate why syncytial knots were recorded in both cases and controls. Even though the present study recorded syncytial knots in both cases and controls, the number of aggregates of syncytiotrophoblast nuclei creating syncytial knots was substantially higher in SCD placentae than in non-SCD placentae. This finding is in line with a recent study conducted on sickle cell placentae which demonstrated a modest overpopulation of syncytial aggregated nuclei [16], possibly due to the impact of SCD on the human placenta. Pregnancy in women with SCD is associated with an increased risk of complications, derived from increased metabolic demand, clotting proclivity, and vascular congestion/occlusion, which appear to accelerate hypoxia or hypoxic perfusion damage, as shown by the insufficient supply of maternal blood to the placenta, which appears to accelerate the production of syncytial knots [8,17]. Other studies have also shown that the increase in the number of syncytial knots is not the only finding of hypoxia and does not necessarily exist with the other morphological changes of hypoxia [18]. Chronic hypoxia may stimulate the growth of capillaries and increase the mean volume density of syncytial knot as observed in SCD placentae [8]. The notable increase in syncytial knots in SCD placentae observed in this study likely reflects a compensatory mechanism to the chronic hypoxic environment caused by the disease, which emphasizes the role of hypoxia in driving placental histomorphological alterations.

The first steps of placentation take place in a low-oxygen environment. In the third month of human pregnancy, maternal blood exclusively enters the growing placenta, and the diet at this time is histotrophic. The presence of oxidants at the early stages of pregnancy may affect the development of the placenta and foetus, hence a low oxygen supply is needed to protect the developing foetus and the placenta [19]. Vascular endothelial growth factor, placental growth factor, and angiopoietin are involved in the growth and vascularization of the placenta and are responsive to hypoxic hypoxemic stimuli [20,21]. In the present study, a significant increase in the volume density of foetal capillaries was observed in the terminal villi of the SCD placentae compared to the controls. The present findings support those of Stanek [22], who reported an increased vascularization in a study on clinical and placental associations of placental acute, chronic, and acute-on-chronic (overlap) hypoxic lesions. The increase in the formation of foetal capillaries in the SCD placentae compared with the controls might be a result of the vaso-occlusive crises sickle cell pregnant women undergo [22]. The changes observed in the present study might be due to reduced blood supply to the foetus, hence stimulating the formation of foetal capillaries in the terminal villi of the placenta by angiogenesis.

The formation of foetal capillaries increases as gestational age progresses from the first to the second trimester of the placenta [23]. Even though the formation of foetal capillaries increases as gestation progresses, it was observed in the present study to be significantly higher in SCD placentae than in the controls. Increased terminal villi, syncytial knots, fewer Hofbauer cells, and a change in vascular profile are signs of a mature placenta. Some studies have demonstrated a rise in villus cytotrophoblasts, Hofbauer cells, and non-apoptotic syncytial knots; a decrease in the extracellular matrix of chorionic villi; and an increase in villus vascularity [22].

The present study recorded a significant decrease in the volume density of intervillous spaces in SCD placentae compared with those of the controls. This finding aligns with Cordier *et al*. [16] who reported a decrease in intervillous spaces among the SCD placentae compared with non-SCD placentae, potentially due to villous agglutination and focal infarctions leading to reduced intervillous space volume [16]. The reduction of the intervillous spaces in SCD placentae may be due to several factors including; (1) entry of syncytial cells into the stroma at great depths and the eroding of capillary lining, and (2) causing the capillaries to enlarge thereby forming sinusoids [24]. Although sinusoids continue to develop in the second and third trimesters, the intervillous space forms, widens, and receives the mother’s initial arterial blood flow [25,26]. This, however, was not the same as observed in SCD placenta where decreased intervillous spaces were observed. Additional research has shown that in chronic placental infections, there are fewer terminal villi and more phagocytes in the intervillous spaces during delivery, potentially slowing the transport of proteins and nutrients across the placenta [27,28]. Decreased intervillous spaces, villi volume, and reduced villous surface area have been reported by Rainey & Mayhew [29] and is corroborated by the findings of the current study. Reduced intervillous space volume density may lead to intermittent maternal blood loss and placental detachment from the uterine wall [30]. The observed reduction in the intervillous spaces of the SCD placentae might occur as a result of villous congestion due to hypoxic events. The observed increase in syncytial knot formation in SCD placentae compared to controls may lead to reduced intervillous spaces, as these knots can shed into the intervillous spaces.

The present study recorded a higher volume density of syncytial denuded areas in the SCD placentae than non-SCD placentae. Syncytial denudation refers to the shedding or loss of the outermost epithelial layer of the placental chorionic villi, known as the syncytiotrophoblast [31]. Some researchers report that, first trimester chorionic villi have more syncytial knots, fibrinoid necrosis, vasculosyncytial membrane, and cytotrophoblast than second trimester placentae [23]. Syncytial denuded areas have been found in other disease conditions. The finding in this study is consistent with studies on *Plasmodium falciparum* infected placentae which found increased fibrinous necrosis, necrotic regions, and syncytial aggregates along with enhanced inflammatory activity [32]. The present study has demonstrated a distortion of the placental syncytium by SCD. This might have resulted in the high statistically significant volume density of syncytial denuded areas in the SCD placentae compared with those of controls. The syncytium has been described as denuded or necrotic leading to necrotic cell death with extensive syncytial ruptures and possibly villi denudation and total villi integrity degradation [33]. Even though several studies have seen the distortions of the syncytiotrophoblasts in pathological conditions similar to SCD condition, the histomorphology of the placenta can be altered by active infection of the placenta at delivery and has been found to have an association with decreased villous area and vascularity, thickening of the basement membrane, increased syncytial knotting, fibrinoid necrosis, and syncytial damage [33,34].

One limitation of this study is the small sample size, as it was conducted at a single institution, which necessitated grouping all SCD subtypes together. The study also did not account for the potential effects of delivery mode, timing of sample collection, or abnormal hemoglobin traits in the control group. Despite these limitations, the study provides valuable insights into the effects of SCD on placental histomorphology and indicates the need for larger, multi-center studies to confirm these findings and explore these variables further.

## 5. Conclusion

Our research, using stereology, identified an increase in syncytial knots and syncytial denuded areas in SCD placentae. These findings may indicate a heightened hypoxic environment and increased placental stress in SCD placentae. Also, the increased volume density of foetal capillaries in SCD placentae may suggest a compensatory angiogenic response to persistent hypoxia, and the reduction in intervillous gaps seen in SCD placentae may suggest vascular congestion and reduced maternal-fetal exchange. Collectively, these findings shed light on the subtle histological changes associated with SCD in placental pathology, emphasizing the need for improved monitoring and management measures during pregnancy in women with SCD.

## Supporting information

S1 Fig Gestational age. (TIF)

S2 Fig Photograph of human placenta from field examination. (TIF)

S1 Data SCD Placenta Studies_Volume density data_Mumuni et al. (XLSX)

S2 Data SCD Plancenta Studies_Gross morphology_Mumuni et al. (XLSX).
