# Supplementary figures and images for: Histomorphology of placentae of women with sickle cell disease during pregnancy – A case control study
Source: PLoS One. 2025 Feb 24;20(2):e0319011. doi: 10.1371/journal.pone.0319011 (PMC11849815; doi:10.1371/journal.pone.0319011)

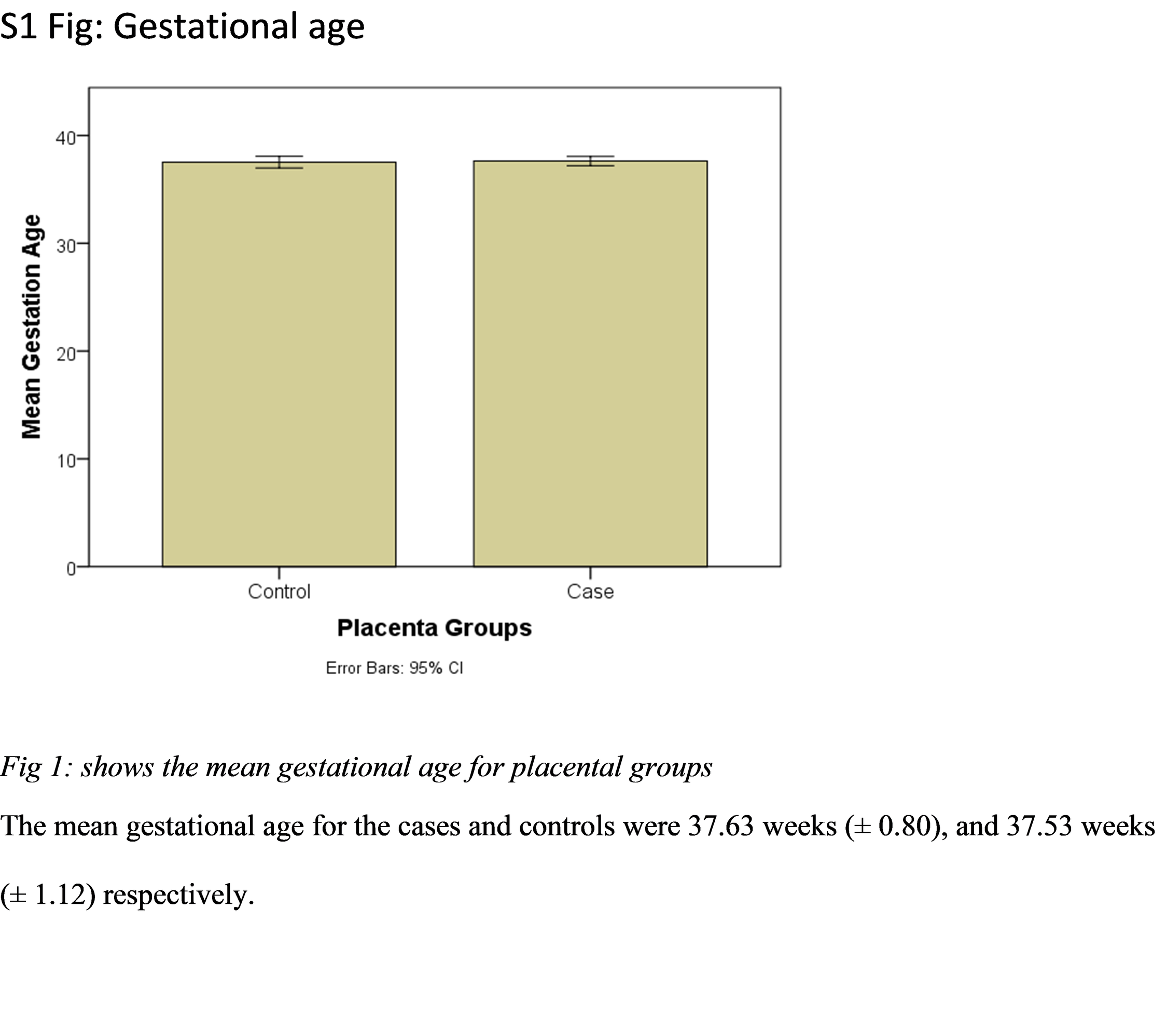

Supplement: S1 Fig — (TIF) [file pone.0319011.s001.tif]

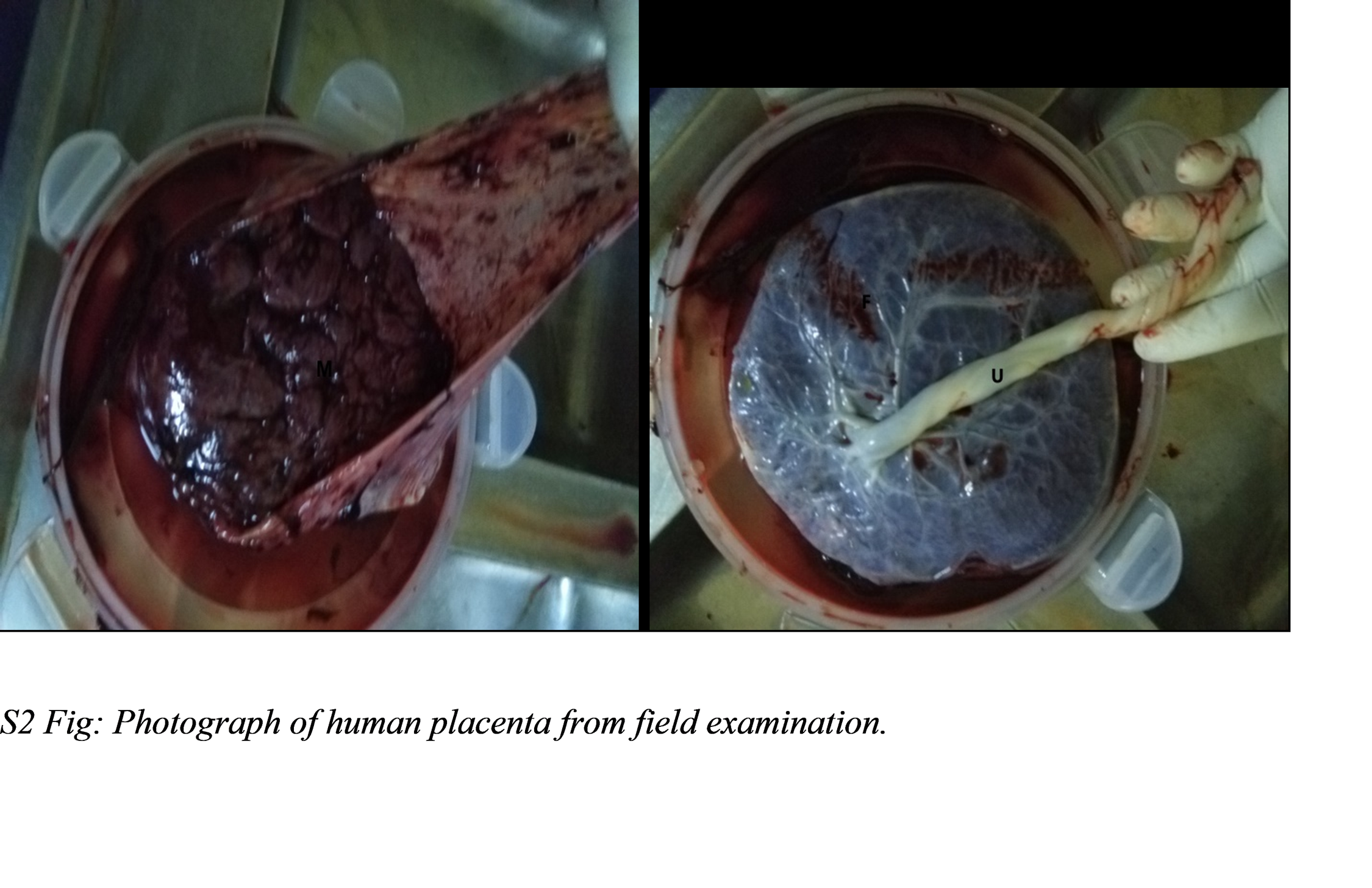

Supplement: S2 Fig — (TIF) [file pone.0319011.s002.tif]
